# Supplementary material for: A Survey of Vaping Use, Perceptions, and Access in Adolescents from South-Central Texas Schools
Source: Int J Environ Res Public Health. 2023 Sep 15;20(18):6766. doi: 10.3390/ijerph20186766 (PMC10530846; doi:10.3390/ijerph20186766)
Supplement: Supplementary file 1 [file ijerph-20-06766-s001.zip › ijerph-2594886-supplementary.pdf]

S1: Data Collection Tool

DEMOGRAPHICS

1. Have you ever completed THIS survey before?
   - Yes (exclude), No
2. How old are you?
   - $\leq$11 (exclude), 12, 13, 14, 15, 16, 17, 18, 19, 20, $\geq$21 (exclude)
3. Are you male or female?
   - Male, Female, Prefer not to say
4. What grade are you in? If Summer, what grade were you in last year?
   - $\leq 5^{th}$ (exclude), 6th, 7th, 8th, 9th, 10th, 11th, 12th, $\geq$ High School Graduate (exclude)
5. Where do you go to school?
   - Middle School South, Middle School North, Voss Middle School, Boerne High, Champion High, Southside Loyola Middle, Southside Losoya Middle, Southside High, Comfort Middle, Comfort High, Other:__________________

Table: Various Activity Perceptions Evaluation (VAPE) Scale

**How healthy or unhealthy do you believe the following activities are?**						
**Activity**	**Very Unhealthy**	**Unhealthy**	**Slightly Unhealthy**	**Slightly Healthy**	**Healthy**	**Very Healthy**
Exercising 30 minutes per day						X (3)
Drinking water					X (2)	
Reading a book				X (1)		
Eating fruits and vegetables					X (2)	
Smoking cigarettes	X (-3)					
Watching movies or television (TV)		X (-2)				
Hanging out with friends			X (-1)			
Walking upstairs or downstairs					X (2)	
Vaping		X (-2)				
Listening to loud music			X (-1)			
Driving in the rain		X (-2)				
Writing in a journal				X (1)		
Drinking alcohol	X (-3)					
Lifting weights						X (3)
Wearing a seatbelt						X (3)
Eating fast food/Drinking soda (Coke, DP)	X (-3)					
Sleeping 7-8 hours per night						X (3)
Texting/E-mailing while driving	X (-3)					
Swimming for fun				X (1)		
Wearing a coat outside in cold weather				X (1)		
Unprotected sex/Multiple partners		X (-2)				
Riding a bike					X (2)	
Skateboarding		X (-2)				
Playing video games		X (-2)				
Attending school					X (2)	
Wearing sunglasses				X (1)		
Wearing a helmet						X (3)
Riding a motorcycle	X (-3)					
Experimenting with drugs/marijuana	X (-3)					
Physical violence/Fist fighting		X (-2)				
Carrying a weapon			X (-1)			
Sunbathing		X (-2)				
Conversing with parents					X (2)	
Attending a concert			X (-1)			
Going to a party			X (-1)			
Riding with driver who has been drinking	X (-3)					
Attending yoga class						X (3)
Meditating					X (2)	
Running or Jogging						X (3)
Having a pet					X (2)	

"X" indicates the predicted value noting parenthetical point assessment values

VAPING SURVEY

1. Do you vape?
   - Yes, No
     - If yes, how many times have you vaped in the past year?
       - ≤5, >5 but <12, ≥1 but <3x Month, ≥4 Month, ≥ 4 Week
     - If yes, how old were you when you first started vaping regularly?
       - Don't regularly, ≤8, 9, 10, 11, 12, 13, 14, 15, 16, 17, 18, 19, 20, ≥21
     - If yes, when you were vaping, have you ever experienced any of the following? (Check all that apply)
       - Shortness of breath/difficulty breathing, coughing/wheezing, anxiety/panic attacks, pain or tightness in the lungs or chest, lightheadedness/fainting, dizziness/loss of balance/foggy feeling, sleepiness, racing heartbeat/pounding feeling in chest or neck, throbbing feeling in head/headache, nausea/vomiting/indigestion, restlessness/hyperactivity/difficulty sleeping/difficulty concentrating, lack of motivation/desire/commitment, difficulty swallowing/dry mouth, anger/frustration/mood swings, sadness/depression, difficulty remembering, fatigue/tired feeling, loss or gain of appetite, blurry vision/difficulty focusing, fever, loss of taste, bloody nose, fast breathing, low blood oxygen level, seizure, Other:______________________
2. Have you ever tried vaping?
   - Yes, No
     - If yes, how old were you the first time you ever tried vaping?
       - ≤8, 9, 10, 11, 12, 13, 14, 15, 16, 17, 18, 19, 20, ≥21
     - If No, do you think you will ever try vaping?
       - Yes, No, Maybe
3. Do you think that vaping and smoking cigarettes is the same thing?
   - Yes, No
     - If no, do you see vaping as tobacco use?
       - Yes, No, Maybe
4. Do you smoke cigarettes?
   - Yes, No
     - If yes, how many times have you smoked in the past year?
       - ≤5, >5 but <12, ≥12 but <3x Month, ≥4 Month, ≥ 4 Week
     - If yes, how old were you when you first started smoking regularly?
       - Don't regularly, ≤8, 9, 10, 11, 12, 13, 14, 15, 16, 17, 18, 19, 20, ≥21
5. Have you ever tried smoking cigarettes?
   - Yes, No
     - If yes, how old were you the first time you ever tried smoking?
       - ≤8, 9, 10, 11, 12, 13, 14, 15, 16, 17, 18, 19, 20, ≥21
     - If No, do you think you will ever try smoking? Yes/No

6. Do you feel UNCOMFORTABLE and want to leave the survey early before finishing?
   - Yes, No
7. Do you think vaping is dangerous?
   - Very dangerous, Dangerous, Slightly dangerous, Slightly safe, Safe, Very Safe
8. How likely are YOU to get sick from regularly vaping? (Please answer even if you do NOT vape)
   - Very unlikely, Unlikely, Slightly unlikely, Slightly likely, Likely, Very likely
9. How likely are OTHERS to get sick from regularly vaping?
   - Very unlikely, Unlikely, Slightly unlikely, Slightly likely, Likely, Very likely
10. Do you think your friend(s) approve or would approve of you vaping?
    - Strongly disapprove, Disapprove, Slightly disapprove , Slightly approve, Approve, Strongly approve
11. Do you think your parent(s) approve or would approve of you vaping?
    - Strongly disapprove, Disapprove, Slightly disapprove, Slightly approve, Approve, Strongly approve
12. How many of your friends do you think vape?
    - 0%, 10%, 20%, 30%, 40%, 50%, 60%, 70%, 80%, 90%, 100%
13. How hard is it for people to vape while at school?
    - Very easy, Easy, Slightly easy, Slightly hard, Hard, Very hard
14. How hard is it for people to vape before or after school?
    - Very easy, Easy, Slightly easy, Slightly hard, Hard, Very hard
15. How hard is it to get a vape, juice, pod, or other vaping supplies?
    - Very easy, Easy, Slightly easy, Slightly hard, Hard, Very hard
16. Where would you go to get a vape or vaping supplies?
    - Friend, Sibling, Parent, Online, Vape Shop, Walmart, Gas Station, Target, Grocery Store, Other
17. How far (from where you live/from your school) do you think you have to go to find the nearest PLACE that sells vapes or vaping supplies?
    - 1-3 blocks, > 3 block < 1 mile, 1-3 miles, 4-6 miles, 7-9 miles, 10-15 miles, 16-25 miles, Over 25 miles
18. How far (from where you live/from your school) do you think you have to go to find the nearest VAPE SHOP?
    - 1-3 blocks, > 3 block < 1 mile, 1-3 miles, 4-6 miles, 7-9 miles, 10-15 miles, 16-25 miles, Over 25 miles

19. Have you ever been in a vape shop?
    - Yes, No
        - If yes, did they check your ID/age when you entered?
            - Yes, No
        - If yes, did a vape shop worker tell you any of the following while you were in the store or at the kiosk? (Check all that apply)
            - Vaping is: safe, rarely the cause of any health problems, NOT addictive, a good way to stop or NOT start smoking, relaxing, full of juice that is pure, clean, or NOT dangerous,
              Other:____
        - If yes, did you buy or try to buy anything?
            - Yes, No
                - If yes, did they check your ID/age at purchase?
                    - Yes, No
        - If yes, have you gone to a vape shop more than once?
            - Yes, No
                - If yes, do they check your ID/age every time you go?
                    - Yes, No
20. How much money do you or would you spend on vaping weekly?
    - $0, $1-10, $11-20, $21-30, $31-40, $41-50, >$50
21. Where do you or would you get the money you spend on vaping?
    - Friend, Sibling, Parent, Grandparents, Job, Other
22. Do you encounter others vaping in public areas where you feel you cannot avoid them (e.g., restrooms, hallways, entrances to buildings)?
    - Yes, No
23. Do laws regarding the minimum age allowed for vaping discourage you from vaping?
    - Yes, No
24. Have you ever discussed in a classroom setting as part of a formal lesson plan or curriculum vaping in school?
    - Yes, No
25. Do you take Public Service Announcements regarding vaping seriously?
    - Yes, No
        - If yes, where is the best place to promote any messaging about the risks of vaping?
            - Social Media, Class, Student Organization, Church Group,
              Other:____
26. Is there something preventing you from learning more about vaping?
    - Yes, No
        - If yes, what do you feel is preventing you from learning more about vaping?
            - Vaping is not dangerous, There are no courses offered at my school, I am vaping to help quit smoking cigarettes, I know vaping can be dangerous but I do NOT want to quit, I do not know where to go to find out about vaping,
              Other:____

VAPING KNOWLEDGE ASSESSMENT

1. Vaping is dangerous and should be avoided?
    a. True
    b. False
2. Vaping can cause any or all of the following health problems:
    a. Difficulty concentrating or making it hard to focus thinking
    b. Difficulty breathing or shortness of breath
    c. Difficulty for the heart to move blood properly or feeling pulse while resting
    d. Difficulty with mood or feeling sad/unhappy
    e. All of the above
3. Texas currently:
    a. Requires public schools to teach vaping health education
    b. Has a complete vaping teaching schedule and materials for health educators to use in class
    c. Has a group of people within the education agency considering vaping health education
    d. Does not have any state leaders concerned about vaping
4. Vaping can cause severe illness in people in just a few weeks.
    a. True
    b. False
5. Vaping has not really ever hurt anyone and is a safe alternative to smoking cigarettes.
    a. True
    b. False
6. Vape juice might have stuff in it that could hurt my lungs or cause me to get sick.
    a. True
    b. False
7. Vape juice is safe as long as it tastes good like fruit or candy.
    a. True
    b. False
8. No one has ever gotten sick from vaping.
    a. True
    b. False
9. What do you think is in vapes?
    a. Just flavoring
    b. Nicotine
    c. Marijuana/THC
    d. Tobacco
    e. All of the above
    f. Unknown

10. What is the worst thing that could happen to someone who vapes?
    a. They have their lungs removed and replaced
    b. They cough
    c. They feel tired, run down, or have difficulty concentrating
    d. They have chest pain or feel a fast heartbeat
    e. They could die
    f. All of the above

DEMOGRAPHICS 2

1. Does anyone in your house vape or smoke?
    - Yes, No
        - o If yes, how often do you think they smoke or vape?
            - Rarely, Sometimes, Frequently, Always
        - o If yes, which type of things do they smoke or vape?
            - (Check All That Apply) cigarettes, vape, cigars, pipe, hookahs, dip, snuff, chewing tobacco, marijuana, other drugs
        - o If yes, how often per day are you able to smell them smoking or vaping?
            - none, <1 hour, 1-2 hours, >2 hours
2. What are your average grades?
    - As, As and Bs, Bs, Bs and Cs, Cs, Cs and Ds, Ds, Ds and Fs, Fs
3. How would you describe yourself?
    - non-Hispanic White, non-Hispanic Black, Hispanic, Asian, Native America/Islander, Two or More, Other/Unknown
4. What language is most often used in your house?
    - English, Spanish, Portuguese, French, German, Mandarin, Arabic, Other
5. Do you live with both of your parents?
    - Both Parents, Single-mom, Single-dad, Grandparent(s), Aunt/Uncle, Guardian, On your own, Foster care
6. How many people living in your house have a job?
    - None, 1, 2, 3, 4, 5, 6, $\geq$7
7. How much money do the adults in your house make combined per year?
    - Unknown, <$25,000, $25,000 to <$50,000, $50,000 to <$100,000, $100,000-200,000, >$200,000
8. What is the highest level of education completed by someone living in your house?
    - <high school, high school/GED, some college, associate degree, bachelor's degree, master's degree, doctorate degree
9. Do you have brothers or sisters living with you?
    - Yes, No
        - o If yes, how many older brothers and/or sisters?
            - 1, 2, 3, 4, 5, 6, $\geq$7
        - o If yes, how many younger brothers and/or sisters?
            - 1, 2, 3, 4, 5, 6, $\geq$7

10. Do more adults than your parents or guardian live in your house?

- Yes, No
    - If yes, how many adults live in your house?
        - 2, 3, 4, 5, 6, ≥7
    - If yes, what relationship do they have to you?
        - (Check All That Apply) grandparent(s), Aunt(s), Uncle(s), Friend(s), Older Brother(s)/Sister(s)
